# Supplementary material for: Identification of photoperiod-induced specific miRNAs in the adrenal glands of Sunite sheep (Ovis aries)
Source: Front Vet Sci. 2022 Jul 22;9:888207. doi: 10.3389/fvets.2022.888207 (PMC9354845; doi:10.3389/fvets.2022.888207)
Supplement: Supplementary file 1 [file Data_Sheet_1.ZIP › Supplementary file/miRDeep2 file.zip!/miRDeep2 file/Know_Structure/LP42A1/oar-mir-665.pdf]

miRBase precursor : oar-mir-665
Total read count : 55
oar-miR-665-5p read count : 2
oar-miR-665-3p read count : 51
remaining reads : 2

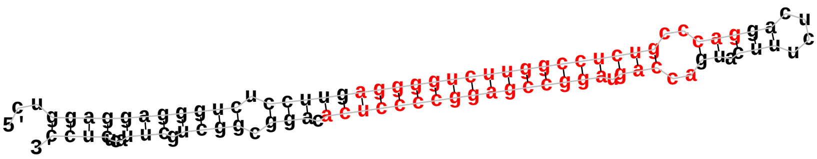

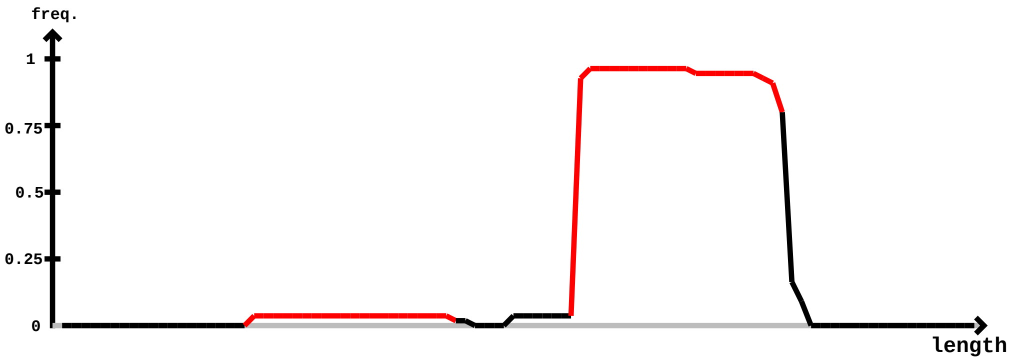


```
                                                                        oar-miR-665-3p
                     oar-miR-665-5p
5'- cuggaggagggucuccuugaggggucuuggccucugcccaggacuuucaugaccaguaggccgaggccccucacaggcggcugcuuacucucc   -3'   exp
    ..((((((((((.((((((((((((((((((((((..(((((....))).))..))).))))))))))))))).))).)))).))).))))..    reads   mm    sample
    ...................aggggucuuggccucugccca.....................................................    1       0     seq
    ...................aggggucuuggccucugcccagg...................................................    1       0     seq
    ............................................uuucaugaccaguaggccg..............................    1       0     seq
    ............................................uuucaugaccaguaggccgaggccccuc.....................    1       0     seq
    ...................................................accaguaggccgaggcccc.......................    1       0     seq
    ...................................................accaguaggccgaggccccu......................    1       0     seq
    ...................................................accaguaggccgaggccccuc.....................    5       0     seq
    ...................................................accaguaggccgaggccccuca....................    35      0     seq
    ...................................................accaguaggccgaggccccucac...................    2       0     seq
    ...................................................accaguaggccgaggccccucaca..................    5       0     seq
    ....................................................ccaguaggccgaggccccucac...................    2       0     seq
```
